# The role of Klotho and FGF23 in cardiovascular outcomes of diabetic patients with chronic limb threatening ischemia: a prospective study

**DOI:** 10.1038/s41598-023-33190-3

**Published:** 2023-04-15

**Authors:** Federico Biscetti, Maria Margherita Rando, Andrea Leonardo Cecchini, Maria Anna Nicolazzi, Enrica Rossini, Flavia Angelini, Roberto Iezzi, Luis H. Eraso, Paul J. Dimuzio, Dario Pitocco, Antonio Gasbarrini, Massimo Massetti, Andrea Flex

**Affiliations:** 1 Cardiovascular Internal Medicine, Department of Cardiovascular Sciences, Fondazione Policlinico Universitario A. Gemelli IRCCS, Largo Agostino Gemelli 8, 00168 Rome, Italy; 2 Università Cattolica del Sacro Cuore, Largo Francesco Vito 1, 00168 Rome, Italy; 3 Radiology Unit, Fondazione Policlinico Universitario A. Gemelli, IRCCS, Rome, Italy; 4 Division of Vascular and Endovascular Surgery, Thomas Jefferson University, Philadelphia, PA USA; 5 Diabetology Unit, Fondazione Policlinico Universitario A. Gemelli, IRCCS, Rome, Italy; 6 Department of Medical and Surgical Sciences, Fondazione Policlinico Universitario A. Gemelli IRCCS, Largo Agostino Gemelli 8, 00168 Rome, Italy; 7 Department of Cardiovascular Sciences, Fondazione Policlinico Universitario A. Gemelli IRCCS, Largo Agostino Gemelli 8, 00168 Rome, Italy

**Keywords:** Cardiovascular diseases, Diabetes

## Abstract

Cardiovascular complications after lower extremity revascularization (LER) are common in diabetic patients with peripheral arterial disease (PAD) and chronic limb threatening ischemia (CLTI). The Klotho-fibroblast growth factor 23 (FGF23) axis is associated with endothelial injury and cardiovascular risk. We aimed to analyze the relationship between Klotho and FGF23 serum levels and the incidence of major adverse cardiovascular events (MACE) and major adverse limb events (MALE) after LER in diabetic patients with PAD and CLTI. Baseline levels of Klotho and FGF23, and their association with subsequent incidence of MACE and MALE were analyzed in a prospective, non-randomized study in a population of diabetic patients with PAD and CLTI requiring LER. A total of 220 patients were followed for 12 months after LER. Sixty-three MACE and 122 MALE were recorded during follow-up period. Baseline lower Klotho serum levels (295.3 ± 151.3 pg/mL vs. 446.4 ± 171.7 pg/mL, *p* < 0.01), whereas increased serum levels FGF23 (75.0 ± 11.8 pg/mL vs. 53.2 ± 15.4 pg/mL, *p* < 0.01) were significantly associated with the development of MACE. Receiver operating characteristic (ROC) analysis confirmed the predictive power of Klotho and FGF23 baseline levels. Furthermore, decreased Klotho levels were associated with the occurrence of MALE after LER (329.1 ± 136.8 pg/mL *vs* 495.4 ± 183.9 pg/mL, *p* < 0.01). We found that Klotho and FGF23 baseline levels are a potential biomarker for increased cardiovascular risk after LER in diabetic patients with PAD and CLTI.

## Introduction

Peripheral arterial disease (PAD) is a common complication of type 2 diabetes mellitus (T2DM) and an independent cardiovascular risk factor^1^. Diabetic patients with PAD have poorer quality of life and life expectancy than patients without PAD. In general, PAD is also more aggressive in people with T2DM than in people without T2DM leading to a higher incidence of tissue loss and amputations^2–4^. One of the most common complications of PAD is chronic limb threatening ischemia (CLTI), which necessitates endovascular revascularization and often results in lower extremity gangrene requiring amputation^5^. Patients with CLTI may also experience major adverse cardiovascular events (MACE) and major adverse limb events (MALE) in the period following revascularization procedure^6^. This phenomenon is primarily due to the fact that atherosclerotic disease in patients with PAD is essentially a polyvascular disease that affects multiple arterial beds in the lower extremities, coronary arteries and brain^7,8^. There is also extensive evidence that other pathophysiological mechanisms involving structural protein of cardiac contractility, atherosclerosis associated inflammation response and platelet activation pathways may play an important role in MACE and MALE after non-cardiac surgical interventions^9,10^. Despite implementation of optimal medical therapy aimed at managing modifiable risk factors including lifestyle modification, use of lowering lipid therapy, use of antiplatelet therapy, novel anticoagulants and medications to control diabetes and high blood pressure^11–13^, still a large number of patients with PAD undergoing revascularization experience MACE and MALE^1^. In this context, identification of novel pathophysiological pathways as adjuvant risk stratification and/or therapeutic targets is important in further reducing the incidence of MACE and MALE on PAD patients CLTI undergoing revascularization for limb salvage.

Klotho, a protein that exists in two forms, membrane-bound Klotho and soluble Klotho, and acts as co-receptor for fibroblast growth factor 23 (FGF23), has been shown to be involved in atherosclerosis and cardiovascular disease^14^. Moreover, Klotho regulates blood glucose and cholesterol levels^15,16^. Furthermore, the lack of Klotho has been found to promote calcification and accumulation of cholesterol in the arteries, which can lead to coronary heart disease^17^. Interestingly, reduced Klotho serum levels are associated with endothelial dysfunction, oxidative stress, accelerated atherosclerosis, plaque instability with increase in atherosclerosis mediated inflammatory response playing a plausible role in acute cardiovascular^14,18–21^. Despite their structural differences, membrane-bound and soluble Klotho are closely related and share several functional properties^18^.

Both Klotho protein forms can act as the obligate co-receptor FGF23^22^, a relatively new member of the fibroblast growth factor family, and has been shown to play a role in the development of atherosclerosis and cardiovascular disease^23,24^. In fact, in individuals with chronic kidney disease (CKD), FGF23 is associated with endothelial dysfunction, arterial wall calcification, left ventricular hypertrophy, coronary artery disease and cardiovascular mortality^23,25,26^. More recently, elevated FGF23 levels have been shown to be associated with the presence and severity of PAD in a diabetic patient population without CKD^24^.

Given the available data, we hypothesized that Klotho and FGF23 baseline levels might influence the incidence of cardiovascular complications after endovascular revascularization of the lower limb (LER).

## Methods

### Study design

The aim of this study was to evaluate the relationship between Klotho and FGF23 serum levels at moment of LER and the incidence of MACE and MALE in a cohort of T2DM patients with PAD and CLTI. We conducted a prospective, non-randomized study approved by the Ethics Committee of the Fondazione Policlinico Universitario A. Gemelli (Istituto di Ricovero e Cura a Carattere Scientifico—IRCCS). All included patients gave informed consent to participate in the study, in accordance with the principles of the Declaration of Helsinki.

### Study population and clinical assessment

The study population included 220 T2DM patients with PAD and CLTI requiring revascularization from the Fondazione Policlinico Universitario A. Gemelli IRCCS in Rome, Italy. Patients were consecutively enrolled between December 20, 2019 and June 30, 2021.

Inclusion criteria included: age at least 18 years, diagnosed with T2DM for at least 1 year, ankle/brachial index (ABI) less than 0.9, at least one lower extremity artery stenosis greater than 50% detected by ultrasound (US) color Doppler, stage 4 or 5 PAD diagnosis according to Rutherford classification, presence of CLTI and LER indication for target artery stenosis, as previously described^6,27–30^. Exclusion criteria were: LER within the last 3 months, diabetic foot ulcer with active infection or osteomyelitis, diabetic peripheral neuropathy, homozygous familial hypercholesterolemia, absolute contraindications to antiplatelet therapy, thrombophilia, anemia severe requiring blood transfusion, active cancer, active autoimmune disease, B or C stage liver disease according to Child–Pugh classification, life expectancy less than 12 months, pregnancy.

To classify and stratify patients with diabetic foot ulcers, the Wound, Ischemia, foot Infection (WIfI) classification system was used. If necessary, radiographic studies to rule out osteomyelitis were performed. Diabetic peripheral neuropathy was excluded as previously described^6^. PAD was defined according to the criteria of the Society for Vascular Surgery and the International Society for Cardiovascular Surgery^28^. All patients underwent lower extremity US. Ultrasound evaluation has also been used to confirm significant stenosis in the setting of arterial calcification in patients with an ABI of 1.40 or higher.

For all patients the following clinical and laboratory data were collected. Complete clinical history, including but not limited to coronary heart disease (CAD), cerebrovascular disease (CVD) history, hypertension, smoking status, body mass index (BMI), blood tests, as described below.

All patients were taking lipid-lowering therapy to achieve a low-density lipoprotein cholesterol (LDL-C) target of less than 55 mg/dL.

At time of revascularization, all patients received single antiplatelet therapy and dual antiplatelet therapy (DAPT) for 1 month after revascularization.

### Endovascular revascularization procedure and follow-up

LER was performed as previously described^6,27,28^. Angioplasty and, if necessary, arterial stenting were defined as successful if the remaining arterial stenosis was less than 30% of the lumen^6^. We excluded from follow-up 19 (7.94%) of 239 patients due to primary treatment failure after revascularization. No major perioperative complications were recorded, as defined by the Society of Interventional Radiology^31^. During the 12-month follow-up period, patients were evaluated at 1, 3, 6 and 12 months after LER to assess the incidence of MACE and MALE outcomes. MACE was defined as a combination of myocardial infarction, stroke and cardiovascular death. MALE refer to the composite of acute limb ischemia, major vascular amputations, limb-threatening ischemia leading to urgent revascularization^6^.

### Blood test and biochemical analysis

On the day of LER, blood samples were collected from all patients after an overnight fast. Glucose, creatinine, calcium, phosphorus, vitamin D, total cholesterol, LDL-C, triglycerides and glycated hemoglobin were assessed. Renal function was calculated by the modification of diet in renal disease (MDRD) formula to define estimated glomerular filtration rate (eGFR)^32^. Serum was separated from blood samples by centrifugation and stored at -80 °C prior to each assessment. Two commercially available ELISA kits (EH3058 and EH4278 from Wuhan Fine Biotech Co.) were used to determine Klotho and FGF23 levels, according to the manufacturers' protocol. The intra- and inter-assay coefficients of variation were 3.5% and 10.5%, respectively. Sensitivity, defined as the mean ± 3 SD of the 0 standard, was calculated as 0.15 pmol/mL. Serum levels were measured twice for each patient and the results were averaged.

### Statistical analysis

Demographic and clinical data were summarized as means (standard deviations) for continuous variables and counts (percentages) for categorical variables. Where appropriate, chi-square and t-tests were used to compare groups. A logarithmic transformation was applied to not normally distributed variables before performing other analyses. Where appropriate, Klotho and FGF23 baseline levels were compared using Mann–Whitney, Kruskal–Wallis and Dunn's multiple comparisons. Multivariate stepwise logistic regression analyses adjusted for traditional atherosclerotic risk factors and Klotho and FGF23 baseline levels were performed. We calculated the area under the receiver operating characteristic (ROC) curve to test the predictive discrimination of MACE or MALE. We elaborated two additional ROC curves for a model including only traditional risk factors [age, sex, BMI, high blood pressure, diabetes duration, smoking status, Rutherford staging, previous cardiovascular and cerebrovascular events, total cholesterol, LDL cholesterol, triglycerides, fasting blood glucose (FBG), HbA1c] (Model 1) and for a model including all the risk factors plus all two proteins as continuous variables (Model 2). We then compared the areas under the ROC curves using the roccomp function in Stata. All analyses were performed using STATA version 14.0 for MacOS (Statistics/Data Analysis, Stata Corporation) and GraphPad Prism version 9.4.0 for MacOS (GraphPad Software, Inc.). Statistical significance was established at *p* < 0.05.

### Ethics approval and consent to participate

The study was approved by the Ethics Committee of the Fondazione Policlinico Universitario A. Gemelli IRCCS and adhered to the principles of the Declaration of Helsinki. All the individuals agreed to participate in the study and gave informed consent.

## Results

### Characteristics of the study population

A total of 220 patients were followed until the end of the study. Of these, 150 were male (68.2%). The average age was 71.3 ± 9.2 years. The median duration of diabetes was 12.4 ± 4.6 years. The mean BMI was 29.5 ± 3.0 kg/m^2^. In total, we enrolled 61 (27.7%) active smokers, 68 (30.9%) former smokers and 91 (41.4%) never smokers. Considering other cardiovascular risk factors, 120 (54.5%) patients had arterial hypertension, 100 (45.4%) patients had hyperlipidemia, 102 (46.4%) patients had a history of CAD and 45 (20.4%) patients had a history of CVD. Looking specifically at the characteristics of PAD, the mean ABI score was 0.39 ± 0.1 and the Rutherford staging included 121 (55.0%) category 4 patients and 99 (45%) category 5 patients. Mean glycated hemoglobin was 8.8 ± 1.5%, LDL-C was 100.7 ± 19.4 mg/dL and mean eGFR was 64.4 ± 14.6 mL/min/1.73m^2^. The mean levels of FGF23 and Klotho were 59.4 ± 17.5 pg/mL and 403.1 ± 179.3 pg/mL, respectively. The full characteristics of the population are shown in Table 1.

**Table 1:** Demographic characteristics and clinical data of the study cohort at baseline.

Number of patients	220
Men/female, n	150:70
Age, years ± SD	71.3 ± 9.2
Diabetes duration, years ± SD	12.4 ± 4.6
BMI, Kg/m^2^ ± SD	29.5 ± 3.0
Smoking (current), n (%)	61 (27.7)
Smoking (former), n (%)	68 (30.9)
Never smoked, n (%)	91 (41.4)
Hypertension, n (%)	120 (54.5)
Hypercholesterolemia, n (%)	100 (45.4)
CAD, n (%)	102 (46.4)
CVD, n (%)	45 (20.4)
Lipid-lowering agent	178 (80.9)
ACE inhibitor or ARB	165 (75.0)
ABI, ± SD	0.39 ± 0.1
Rutherford II-4, n (%)	121 (55.0)
Rutherford III-5, n (%)	99 (45.0)
WIfI010, n (%)	99 (45.0)
WIfI020, n (%)	35 (15.9)
WIfI110, n (%)	62 (28.2)
WIfI120, n (%)	107 (48.6)
HbA1c, % ± SD	8.8 ± 1.5
FBG, mg/dL ± SD	114.1 ± 19.8
Total cholesterol, mg/dL ± SD	204.1 ± 29.3
LDL cholesterol, mg/dL ± SD	100.7 ± 19.4
Triglycerides, mg/dL ± SD	212.2 ± 36.9
Creatinine, mg/dL ± SD	1.4 ± 0.4
eGFR, mL/min/1.73m^2^ ± SD	64.4 ± 14.6
Ca, mg/dL ± SD	9.5 ± 0.8
Ph, mg/dL ± SD	3.7 ± 0.8
Vitamin D, ng/mL ± SD	48.5 ± 15.9
Klotho, pg/mL ± SD	403.1 ± 179.3
FGF23 pg/mL ± SD	59.4 ± 17.5

Data are reported as means (standard deviation) for continuous variables and numbers (percentages) for categorical variables. *BMI* Body Mass Index; *CAD* Coronary Artery Disease; *CVD* Cerebrovascular Disease; *ACE* angiotensin-converting enzyme; *ARB* angiotensin receptor blocker; *ABI* Ankle Brachial Index; *WIfI* Wound, Ischemia, foot Infection; *FBG* Fasting Blood Glucose; *eGFR* estimated Glomerular Filtration Rate; *Ca* Calcium; *Ph* Phosphorus; *FGF23* Fibroblast Growth Factor 23.

### Serum levels of Klotho and FGF23 and incidence of MACE at 12 months

All patients were followed up for 12 months. During follow-up, we observed 63 MACEs. The first MACE after LER occurred on day 23 and the last MACE occurred on day 360 after procedure. The mean time of occurrence of MACE was after 193 days.

MACE patients were exclusively male (63, *p* < 0.01), were predominantly suffering from hypertension (46, *p* < 0.01) and were active (30, *p* < 0.01) or ex-smokers (23, *p* < 0.01). There were no significant differences in history of hypercholesterolemia (*p* = 0.62), previous CAD (*p* = 0.06), previous CVD (*p* = 0.49) or LDL levels (*p* = 0.50) between people with and without MACE. Furthermore, we did not find any significant differences in diabetes duration (*p* = 0.62), FBG (*p* = 0.27), HbA1c levels (*p* = 0.08) or eGFR (*p* = 0.93) between the two populations. We documented that serum calcium levels in patients with MACE were slightly higher, but within the normal range (9.7 ± 0.7 mg/dL *vs* 9.4 ± 0.8 mg/dL, *p* = 0.03).

**Figure 1:**
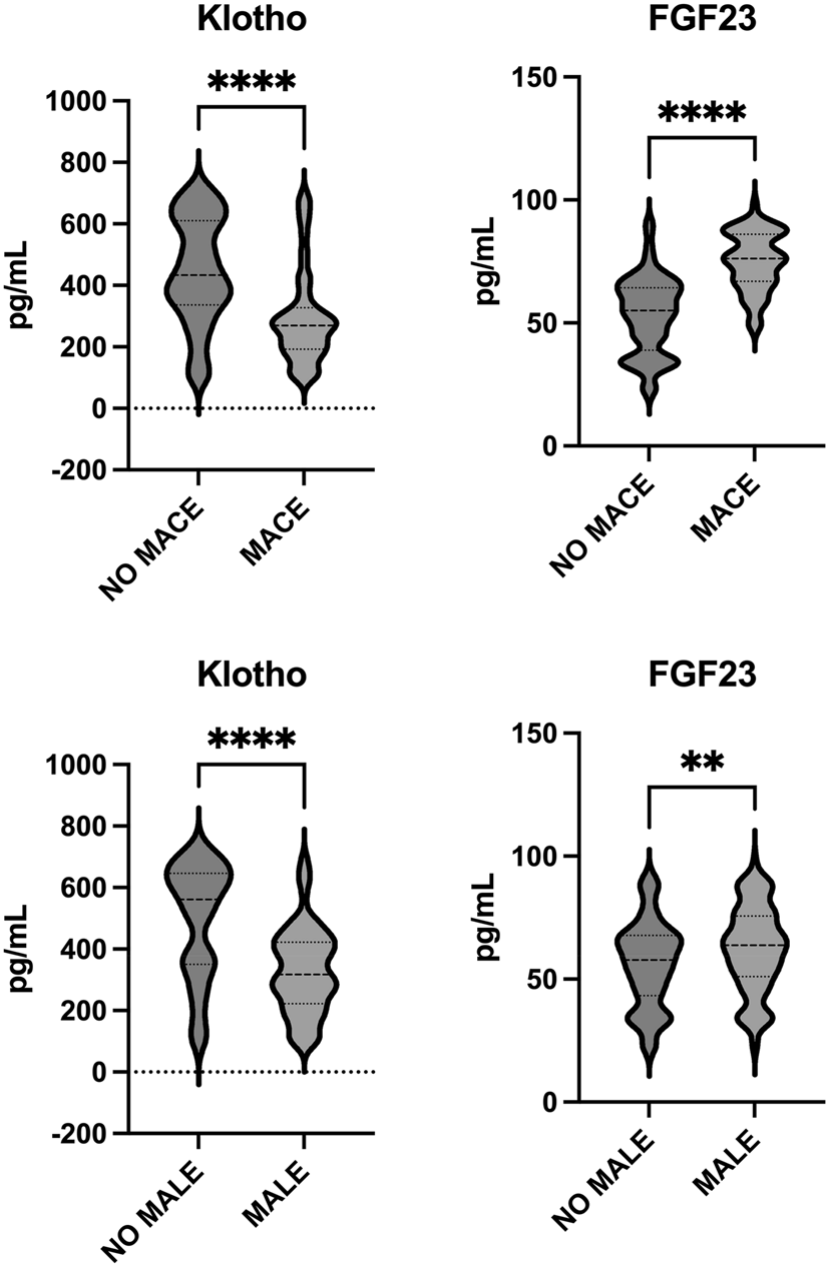
Klotho, and FGF23 levels according to MACE and MALE outcomes. On the violin plots, shape shows the distribution, central line represents the median, upper line represents the upper interquartile range (IQR) and the lower line represents the lower IQR. **** = *p* < 0.0001, ** = *p* < 0.01.

We found higher baseline levels of FGF23 (75.0 ± 11.8 pg/mL *vs* 53.2 ± 15.4 pg/mL, *p* < 0.01) and lower baseline levels of Klotho (295.3 ± 151.3 pg/mL *vs* 446.4 ± 171.7 pg/mL, *p* < 0.01) in MACE patients. Supplemental Table 1 and Fig. 1 provide a comprehensive overview of the characteristics of patients with and without MACE.

We then considered the three components of the MACE composite outcome (Supplemental Fig. 1). Considering cardiovascular death, we did not notice any differences in terms of FGF23 (*p* = 0.12) and Klotho (*p* = 0.48) levels. We found higher baseline levels of FGF23 (*p* < 0.01) and lower baseline levels of Klotho (*p* < 0.05) in CAD patients. We also recorded higher levels of FGF23 (*p* < 0.01) and lower levels of Klotho (*p* < 0.01) in CVD patients at the moment of LER.

**Figure 2:**
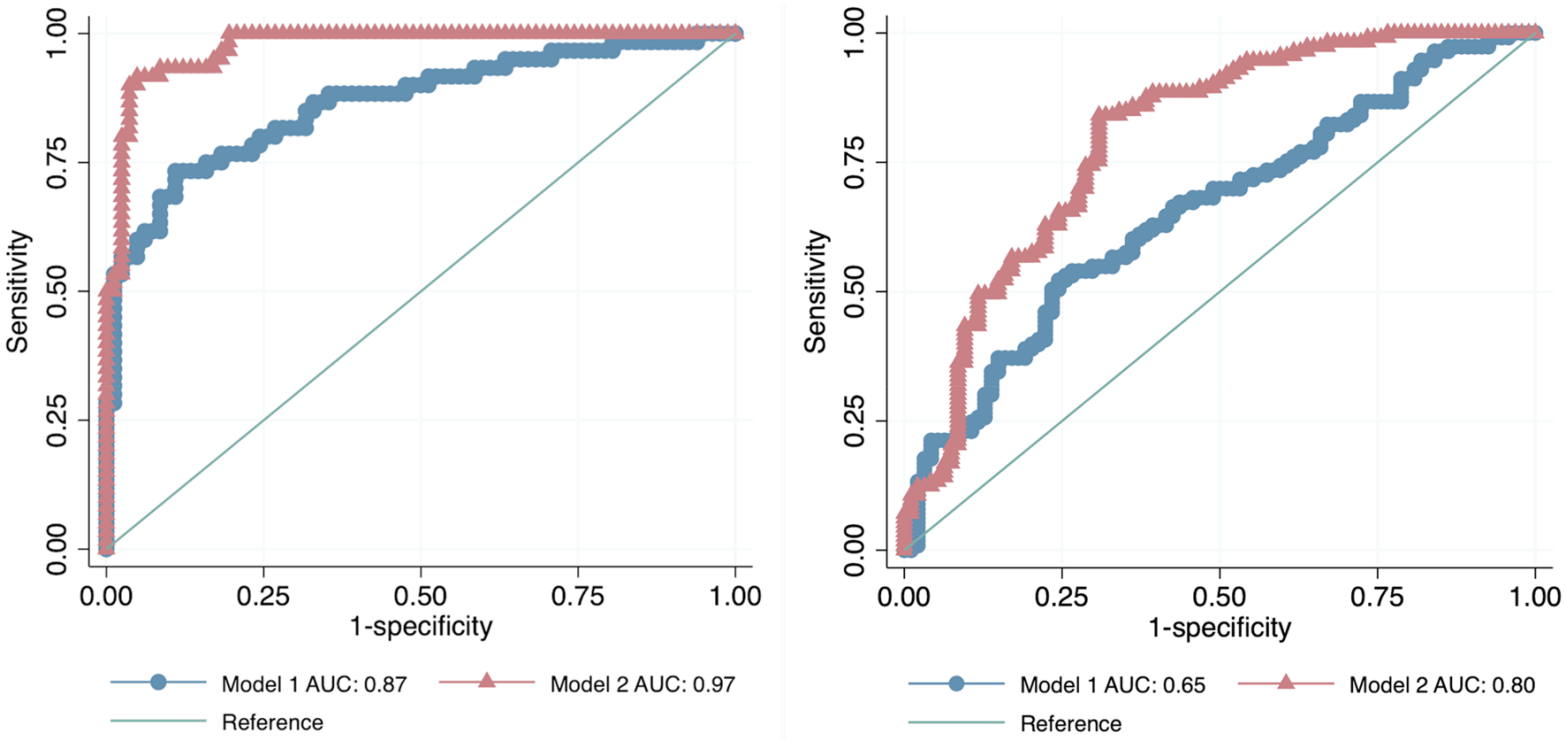
On the left, receiver operating characteristic (ROC) curves comparing the performance of a model without (Model 1) and with proteins (Model 2) in predicting MACE. The true-positive rate (sensitivity) is plotted as a function of the false-positive rate (1—Specificity). *p* < 0.001. On the right, receiver operating characteristic (ROC) curves comparing the performance of a model without (Model 1) and with proteins (Model 2) in predicting MALE. The true-positive rate (sensitivity) is plotted as a function of the false-positive rate (1—Specificity). *p* < 0.001.

Two ROC curves were constructed to predict the incidence of MACE based on Klotho and FGF23 baseline levels and the areas under the curve (AUC) were 0.24 [95% confidence interval (CI) 0.17, 0.32] and 0.87 (95% CI 0.82, 0.92), respectively (Supplemental Fig. 2). To test the efficacy of knowing protein levels at baseline, we compared the predictive power of traditional risk factors and risk factors plus Klotho and FGF23 levels at the moment of the revascularization. Including serum protein levels significantly improved the prediction of incident MACE after LER (Fig. 2).

Multivariate analysis showed that Klotho (*p* < 0.01) and FGF23 (*p* < 0.01) baseline levels were independent determinant of MACE in CLTI patients receiving LER (Table 2). An additional multivariate analysis was performed including all the variables and we found that male sex (*p* < 0.01), BMI (*p* < 0.05), hypertension (*p* < 0.05) and smoking status (current or former, *p* < 0.01) were independent determinant of MACE. Notably, after adjustment for all traditional risk factors, Klotho (*p* < 0.01) and FGF23 (*p* < 0.01) levels were still independent determinant of MACE (Table 3).

**Table 2:** Multivariable logistic regression for MACE.

	Coef	St.Err	t-value	*p*-value	[95% Conf	Interval]	Sig
Klotho	− .001	0	− 4.50	0	− .001	0	**
FGF23	.013	.001	9.06	0	.01	.016	**
Constant	− .243	.12	− 2.03	.044	− .478	− .007	*
Mean dependent var		0.290		SD dependent var		0.455	
R-squared		0.395		Number of obs		207	
F-test		66.513		Prob > F		0.000	
Akaike crit. (AIC)		162.321		Bayesian crit. (BIC)		172.319	

** p < .01, * p < *.05.*

**Table 3:** Multivariable logistic regression for MACE.

	Coef	St.Err	t-value	*p*-value	[95% Conf	Interval]	Sig
Age	.002	.002	0.91	.366	− .003	.007	
Male sex	.187	.051	3.70	0	.087	.287	**
BMI	− .019	.008	− 2.27	.025	− .036	− .002	*
Diabetes duration	.003	.005	0.55	.586	− .007	.012	
Hypertension	.108	.052	2.10	.038	.006	.21	*
Hypercholesterolemia	− .006	.045	− 0.13	.895	− .095	.083	
CAD	− .079	.045	− 1.74	.084	− .168	.011	
CVD	− .047	.066	− 0.71	.481	− .178	.084	
Smoking (current)	.176	.054	3.26	.001	.069	.282	**
Smoking (former)	.289	.048	5.99	0	.194	.384	**
Never smoked	.108	.074	1.46	.145	− .038	.254	
ABI	− .832	1.143	− 0.73	.467	− 3.087	1.423	
Rutherford II-4	0						
Rutherford III-5	.162	.101	1.60	.111	− .038	.363	
WIfI010	0						
WIfI020	.062	.096	0.65	.515	− .126	.251	
WIfI110	.068	.139	0.49	.623	− .206	.342	
WIfI120	.107	.209	0.51	.608	− .304	.519	
LDL cholesterol	.001	.001	0.45	.65	− .002	.003	
FPG	− .002	.001	− 1.38	.169	− .004	.001	
HbA1c	.019	.015	1.22	.223	− .011	.049	
eGFR	− .001	.001	− 0.48	.632	− .004	.002	
Ca	.016	.028	0.56	.573	− .04	.071	
Ph	.001	.027	0.02	.982	− .054	.055	
Vitamin D	0	.001	− 0.22	.825	− .003	.002	
Klotho	− .001	0	− 3.87	0	− .001	0	**
FGF23	.009	.001	6.46	0	.006	.012	**
Constant	.058	.569	0.10	.919	− 1.065	1.182	
Mean dependent var		0.290	SD dependent var		0.455		
R-squared		0.656	Number of obs		207		
F-test		13.827	Prob > F		0.000		
Akaike crit. (AIC)		91.146	Bayesian crit. (BIC)		177.797		

** p < .01, * p < *.05.*

### Serum levels of Klotho and FGF23 and incidence of MALE at 12 months

During the 12 months after LER intervention, we registered 122 MALE.

In Supplemental Table 2 and Fig. 1 are fully reported the characteristics of patients with and without MALE. However, MALE patients were mostly male (90, *p* < 0.05) and suffering from hypertension (74, *p* = 0.04). They mainly were ex-smokers (68, *p* < 0.05) but there were no differences concerning other traditional cardiovascular risk factors. Interestingly, we found higher baseline levels of FGF23 (62.2 ± 17.3 pg/mL *vs* 56.1 ± 17.3 pg/mL, *p* < 0.01) and lower baseline concentrations of Klotho (329.1 ± 136.8 pg/mL *vs* 495.4 ± 183.9 pg/mL, *p* < 0.01) in MALE patients.

We then constructed ROC curves on Klotho and FGF23 baseline levels to predict the incidence of MALE after LER intervention. The two AUC were 0.24 (95% CI 0.17, 0.31) and 0.61 (95% CI 0.53, 0.68) respectively (Supplemental Fig. 2). As for the MACE outcome, we compared ROCs with traditional risk factors alone and with risk factors plus Klotho and FGF23 in predicting MALE. Likewise, addition of baseline protein levels significantly improved the predictive power of MALE after LER (Fig. 2).

Multivariate analysis showed that only baseline Klotho levels (*p* < 0.01) were independent determinants of MALE in CLTI patients during the 12 months after LER (Table 4). A further multivariate analysis, including the other variables, showed that hypertension (*p* < 0.05) and Rutherford stage 5 (*p* < 0.05) persisted to be independent determinants of MALE. Remarkably, after adjusting for all factors, Klotho baseline levels (*p* < 0.01) remained independent determinants of MALE during the follow-up (Table 5).

**Table 4:** Multivariable logistic regression for MALE.

	Coef	St.Err	t-value	*p*-value	[95% Conf	Interval]	Sig
Klotho	− .001	0	− 7.03	0	− .002	− .001	**
FGF23	.002	.002	1.05	.293	− .002	.006	
Constant	.936	.149	6.30	0	.643	1.23	**
Mean dependent var		0.546		SD dependent var		0.499	
R-squared		0.223		Number of obs		207	
F-test		29.237		Prob > F		0.000	
Akaike crit. (AIC)		252.555		Bayesian crit. (BIC)		262.554	

** p < *.01.*

**Table 5:** Multivariable logistic regression for MALE.

	Coef	SE	t-value	*p*-value	[95% Conf	Interval]	Sig
Age	.002	.004	0.48	.63	− .005	.009	
Male sex	.015	.074	0.20	.838	− .13	.16	
BMI	− .01	.012	− 0.83	.41	− .034	.014	
Diabetes duration	.007	.007	1.06	.292	− .006	.021	
Hypertension	.195	.075	2.59	.01	.047	.343	*
Hypercholesterolemia	.04	.065	0.61	.54	− .089	.169	
CAD	− .06	.066	− 0.92	.361	− .19	.07	
CVD	− .11	.096	− 1.14	.254	− .3	.08	
Smoking (current)	− .08	.078	− 1.02	.309	− .235	.075	
Smoking (former)	− .058	.07	− 0.83	.41	− .196	.08	
Never smoked	.015	.108	0.14	.889	− .197	.227	
ABI	2.046	1.661	1.23	.22	− 1.231	5.323	
Rutherford II-4	0						
Rutherford III-5	− .345	.148	− 2.34	.02	− .637	− .054	*
WIfI010	0						
WIfI020	− .15	.139	− 1.08	.283	− .424	.125	
WIfI110	− .043	.202	− 0.21	.83	− .442	.355	
WIfI120	− .247	.303	− 0.82	.416	− .846	.351	
LDL cholesterol	.001	.002	0.69	.49	− .002	.004	
FPG	− .002	.002	− 1.39	.167	− .005	.001	
HbA1c	.002	.022	0.08	.937	− .042	.045	
eGFR	− .001	.002	− 0.41	.684	− .005	.003	
Ca	.065	.041	1.60	.111	− .015	.146	
Ph	.006	.04	0.14	.887	− .073	.084	
Vitamin D	− .001	.002	− 0.25	.805	− .005	.003	
Klotho	− .001	0	− 6.65	0	− .002	− .001	**
FGF23	.002	.002	1.04	.3	− .002	.006	
Constant	− .028	.828	− 0.03	.973	− 1.661	1.606	
Mean dependent var		0.546	SD dependent var	0.499			
R-squared		0.312	Number of obs	207			
F-test		3.276	Prob > F	0.000			
Akaike crit. (AIC)		273.457	Bayesian crit. (BIC)	360.107			

** p < .01, * p < *.05.*

## Discussion

In this prospective study, we demonstrate that altered Klotho and FGF23 baseline levels are associated with higher incidence of adverse cardiovascular outcomes after revascularization in diabetic patients with PAD and CLTI. This is a novel finding, it is consistent with the established role of Klotho-FGF23 axis in atherosclerosis, inflammation and endothelial dysfunction found in other humans and animal models. In particular, decreased Klotho basal serum levels and increased FGF23 levels were associated with the incidence of MACE after revascularization intervention. In addition, decreased Klotho levels were also associated with the development of MALE.

The Klotho gene (also known as α-Klotho) is located on chromosome 13q12 and it was first identified as an anti-aging factor^33^. It is expressed in the kidney and even less in the pancreatic islets, lung, liver, skeletal muscle, aorta, brain and prostate. Klotho exists in two protein forms, membrane-bound and soluble, which play different but both closely related roles and, in particular, both involved as co-receptors of FGF23^22^. In fact, Klotho acts primarily as a coreceptor of FGF23, but also interacts with other receptor systems, including transforming growth factor-β and insulin receptors and signaling pathways, including wingless-related integration site (Wnt)^33^. Klotho is associated to CKD but exerts multiple cardiovascular protective effects due to different receptors and regulatory systems. In fact, it induces Akt expression, through but not only the insulin receptor system, thereby reducing oxidative stress and increasing nitric oxide (NO) production in the endothelium and preventing endothelial dysfunction^34,35^. Furthermore, Klotho deficiency is associated with the development of hypertension, particularly by increasing the stiffness of the arterial walls^36^. Moreover, Klotho and FGF23 are implicated in calcium balance and arterial calcification, as well as other pro-inflammatory mediators, such as osteoprotegerin^37,38^. The Klotho-FGF23 axis is also involved in the development of left ventricular hypertrophy^39^. In fact, Klotho inhibits FGF23- and angiotensin II-induced myocardial hypertrophy^39^. Since these data were primarily obtained in animal models, often genetically modified, it is unclear whether this impairment is due to increased FGF23 activity and/or decreased Klotho function. Nevertheless, both proteins have been shown to play a role in causing the disease. However, soluble Klotho was found to reduce cardiac hypertrophy, independently of FGF23 and phosphate levels, by inhibiting abnormal activity of calcium-dependent signaling in the heart^40^. In addition, Klotho inhibits cardiac remodeling through the Wnt pathway^33^. Interestingly, increased levels of Klotho reduce oxidative damage after myocardial infarction in animal models^41^. Furthermore, reduced Klotho levels are also associated with central obesity, elevated triglycerides and metabolic syndrome^42^. Not surprisingly, reduced Klotho levels and elevated FGF23 concentrations have been associated with increased hospitalizations for heart failure and the incidence of cardiovascular death in a population of 3555 patients with stable ischemic heart disease^43^. The main findings of our study are consistent with previous data and show that reduced Klotho serum levels and elevated FGF23 concentration at the moment of LER are associated with the development of MACE in a T2DM population. Diabetic patients with PAD are frequently affected by polyvascular disease and are at high cardiovascular risk^11,44^.

The prospective nature of the present study, one of the first in humans, provides further insight on potential novel risk factors of MACE after revascularization in patients with CLTI. Traditional risk factors including Male gender, smoking and arterial hypertension were associated to the development of MACE as expected. Remarkably, also calcium levels were slightly, but significantly, elevated in the MACE population. Conceivably, changes in the Klotho/FGF23 serum levels are responsible, at least in part, for altered calcium balance and increased cardiovascular risk. However, multivariate analysis did not confirm a significant role for calcium levels, while it did corroborate that male sex, history of CAD and total and LDL cholesterol levels are crucial determinant for MACE risk. This is consistent with previous data regarding classical cardiovascular risk factors and lipid profile as biomarkers for the MACE incidence^6,7^. As mentioned, Klotho is associated with chronic kidney disease and CKD patients were also included in this study population. However, this did not appear to have influenced the outcomes. Indeed, there were no significant differences in renal function between the different subgroups and eGFR values were not determinant in multivariable logistic regression for MACE and MALE. Smoking status was also relevant. In fact, present and past smoking was significant. Relatedly, even when these factors were included in the analysis model, the results indicated that baseline levels of Klotho and FGF23 were independent determinants of MACE incidence after LER. Interestingly, this hypothesis was also supported by the ROC analysis, with the area under the curve showing the predictive power of the two factors. To our knowledge, this is the first demonstration in prospective human study of a role for the Klotho and FGF23 serum levels at the moment of the revascularization in the development of cardiovascular complications after LER in diabetic patients with PAD and CLTI.

Considering all the possible mechanisms involved, we can assume that the reasons for our finding are multiple and interdependent. Endothelial injury and dysfunction associated with reduced Klotho levels and elevated FGF23 concentrations have been shown to justify the worsening of atherosclerosis in diabetic patients with PAD. Imbalance in calcium metabolism may also be associated with aggravation of arterial wall injury, consistent with data from other proinflammatory cytokines involved in calcium and phosphorus metabolism^6^. In addition, oxidation of LDL may play a role, which may also have therapeutic implications. The study population had no target LDL levels, ​​according to the latest guidelines, and statins have, among other pleiotropic effects, the ability to increase circulating Klotho levels. Conceivably, at least a part of patients, those with reduced Klotho concentrations, may benefit more from statin therapy when reaching the same LDL levels. Furthermore, drugs that inhibit the renin-angiotensin system promote Klotho expression and it is plausible this class of drugs could have better effects in patients who have reduced Klotho serum levels, regardless of the effect on blood pressure. Unfortunately, the relatively small sample size did not allow to stratify the outcomes for the different lipid-lowering or antihypertensive treatments, so we are currently unable to confirm this hypothesis. The small number of individuals studied could also explain the lack of differences observed when analyzing the baseline levels of Klotho and FGF23 in the first component of the composite outcome MACE, specifically in cardiovascular death. However, the significant difference was maintained for CAD and CVD.

Another finding of the present study was the relationship between baseline Klotho levels ​​and the incidence of MALE after revascularization intervention. Considering the characteristics of the population with and without MALE during follow-up, we observed differences in male sex, hypertension, and Klotho and FGF23 baseline levels. Interestingly, as with the MACE outcome, calcium levels were slightly elevated in the MALE population. However, multivariate analysis confirmed that only hypertension and Rutherford stage 5 had a significant role in the development of MALE. Importantly, reduced Klotho levels at the moment of LER were identified as a predisposing factor for MALE incidence even after adjusting for all factors considered in the study. ROC analysis also confirmed the good predictive power of MALE after LER in our patient population. The mechanisms leading to increased MALE in patients with low basal Klotho levels at the time of LER may be diverse, but maybe related to direct endothelial injury, which has been already demonstrated in other models^34,45,46^. Surprisingly, FGF23 baseline levels did not prove to be a significant predictor after inclusion in multivariate analysis. This may be related to the main limitation of the study, namely the relatively small sample size. However, Klotho has been shown to exert a range of endothelial protective effects independent of FGF23 activity, which may justify the effects observed in our study population.

In addition to the relatively small population, limitations of the study include that it was conducted at a single center in a Caucasian population. However, significant differences in Klotho/FGF23 serum levels between races were not described, except that FGF23 levels were associated with food insecurity in a black population^47^. A further limitation is that no hematological data are available to rule out the presence of severe anemia, which could be relevant to the incidence of MACE and MALE. Moreover, the small sample size did not even allow us to stratify the protein levels of the different components of the composite endpoint MALE. Another limitation of our study is that we did not assess the association between original arterial lesions and following incident MALE. Also, we excluded patients with primary LER failure from the study to reduce bias and therefore we did not analyze the serum levels of Klotho and FGF23. Furthermore, we did not measure protein levels during follow-up, and we have no way of knowing whether these changed over time. Nevertheless, the aim of this study was to identify possible biomarkers of risk after LER and further determination over time is not necessary. Finally, we did not analyze the effects of various ongoing pharmacotherapies, especially those that may affect Klotho levels and activity, such as statins, renin‐angiotensin system inhibitors and pioglitazone.

In conclusion, we have demonstrated for the first time that altered Klohto and FGF23 baseline serum levels are associated with the development of MACE after LER and that reduced Klotho baseline levels predict MALE after revascularization. These data were obtained in a relatively small but particularly selected population and need to be confirmed in a larger scenario. Future research will also be able to better elucidate the interactions between Klotho, especially the membrane-bound form, and FGF23 and to highlight whether these effects are independent of each other. It will also be important to know whether current and future therapies can bias the risk from axis imbalance towards, for example, a decrease in Klotho or an increase in FGF23.

Taken together, these data could improve cardiovascular risk stratification in diabetic patients with PAD and help physicians identify personalized treatments.

## Supplementary Information

Supplementary Information.

## Data Availability

The datasets generated during the current study are available from the corresponding author on reasonable request.

## Abbreviations

ABI: Ankle/brachial index
AUC: Area under the curve
BMI: Body mass index
CAD: Coronary artery disease
CI: Confidence interval
CLTI: Chronic limb-threatening ischemia
CVD: Cerebrovascular disease
DAPT: Double antiplatelet therapy
eGFR: Estimated glomerular filtration rate
FBG: Fasting blood glucose
FGF23: Fibroblast growth factor 23
LDL-C: Low-density lipoprotein cholesterol
LER: Lower limb revascularization
MACE: Major adverse cardiovascular events
MALE: Major adverse limb events
NO: Nitric oxide
PAD: Peripheral artery disease
ROC: Receiver operating characteristics
T2DM: Type 2 diabetes mellitus
US: Ultrasound
WIfI: Wound, ischemia, foot infection
Wnt: Wingless-related integration site
