# Supplementary material for: The role of Klotho and FGF23 in cardiovascular outcomes of diabetic patients with chronic limb threatening ischemia: a prospective study
Source: Sci Rep. 2023 Apr 15;13:6150. doi: 10.1038/s41598-023-33190-3 (PMC10105766; doi:10.1038/s41598-023-33190-3)
Supplement: Supplementary file 1 — Supplementary Information. [file 41598_2023_33190_MOESM1_ESM.docx]

**Supplemental Tables**

**Supplemental Table 1. Demographic and clinical data of study participants without or with MACE**

|  | NO MACE (n = 157) | MACE (n = 63) | p value |
| --- | --- | --- | --- |
| Men/female, n | 87:70 | 63:0 | <0.01 |
| Age, years ± SD | 70.8 ± 9.4 | 72.5 ± 8.9 | 0.23 |
| Diabetes duration, years ± SD | 12.5 ± 4.6 | 12.2 ± 4.6 | 0.62 |
| BMI, Kg/m^2^ ± SD | 29.6 ± 2.9 | 29.5 ± 3.3 | 0.85 |
| Smoking (current), n (%) | 31 (19.7) | 30 (47.6) | <0.01 |
| Smoking (former), n (%) | 45 (28.7) | 23 (36.5) | <0.05 |
| Never smoked, n (%) | 81 (51.6) | 10 (15.9) | <0.01 |
| Hypertension, n (%) | 74 (47.1) | 46 (73.0) | <0.01 |
| Hypercholesterolemia, n (%) | 73 (46.5) | 27 (42.8) | 0.62 |
| CAD, n (%) | 72 (45.8) | 30 (47.6) | 0.06 |
| CVD, n (%) | 34 (21.6) | 11 (17.5) | 0.49 |
| ABI, ± SD | 0.39 ± 0.1 | 0.40 ± 0.1 | 0.20 |
| Rutherford II-4, n (%) | 92 (58.6) | 29 (46.0) | 0.09 |
| Rutherford III-5, n (%) | 65 (41.4) | 34 (54.0) | 0.09 |
| WIfI010, n (%) | 29 (18.5) | 6 (9.5) | 0.10 |
| WIfI020, n (%) | 44 (28.0) | 18 (28.6) | 0.93 |
| WIfI110, n (%) | 73 (46.5) | 34 (54.0) | 0.32 |
| WIfI120, n (%) | 11 (7.0) | 5 (7.9) | 0.81 |
| HbA1c, % (mmol/mol) ± SD | 8.7 (72) ± 1.5 | 9.1 (76) ± 1.6 | 0.08 |
| FBG, mg/dL ± SD | 115.0 ± 19.7 | 111.8 ± 20.0 | 0.27 |
| Total cholesterol, mg/dL ± SD | 205.8 ± 30.6 | 199.9 ± 25.6 | 0.17 |
| LDL cholesterol, mg/dL ± SD | 100.2 ± 19.9 | 102.1 ± 18.0 | 0.50 |
| Triglycerides, mg/dL ± SD | 211.7 ± 37.5 | 213.3 ± 35.5 | 0.78 |
| Creatinine, mg/dL ± SD | 1.4 ± 0.4 | 1.4 ± 0.4 | 0.42 |
| eGFR, mL/min/1.73m^2^ ± SD | 64.4 ± 15.2 | 64.3 ± 13.1 | 0.93 |
| Ca, mg/dL ± SD | 9.4 ± 0.8 | 9.7 ± 0.7 | 0.03 |
| Ph, mg/dL ± SD | 3.8 ± 0.8 | 3.6 ± 0.8 | 0.22 |
| Vitamin D, ng/mL ± SD | 48.6 ± 15.6 | 48.4 ± 16.9 | 0.93 |
| Klotho, pg/mL ± SD | 446.4 ± 171.7 | 295.3 ± 151.3 | <0.01 |
| FGF23 pg/mL ± SD | 53.2 ± 15.4 | 75.0 ± 11.8 | <0.01 |

Statistical test performed with Student’s t-test or with Chi square test, when appropriate. BMI, Body Mass Index; CAD, Coronary Artery Disease; CVD, Cerebrovascular Disease; ABI, Ankle Brachial Index; WIfI, Wound, Ischemia, foot Infection; FBG, Fasting Blood Glucose; eGFR, estimated Glomerular Filtration Rate; Ca, Calcium; Ph, Phosphorus; FGF23, Fibroblast Growth Factor 23.

**Supplemental Table 2. Demographic and clinical data of study participants without or with MALE**

|  | NO MALE (n = 98) | MALE (n = 122) | p value |
| --- | --- | --- | --- |
| Men/female, n | 60:38 | 90:32 | <0.05 |
| Age, years ± SD | 71.2 ± 9.1 | 71.3 ± 9.4 | 0.92 |
| Diabetes duration, years ± SD | 12.2 ± 4.9 | 12.7 ± 4.4 | 0.44 |
| BMI, Kg/m^2^ ± SD | 29.6 ± 2.8 | 29.5 ± 3.1 | 0.77 |
| Smoking (current), n (%) | 25 (25.5) | 36 (29.5) | 0.51 |
| Smoking (former), n (%) | 27 (27.5) | 68 (55.7) | <0.05 |
| Never smoked, n (%) | 46 (46.9) | 22 (18.0) | <0.01 |
| Hypertension, n (%) | 46 (46.9) | 74 (60.6) | 0.04 |
| Hypercholesterolemia, n (%) | 43 (43.9) | 57 (46.7) | 0.67 |
| CAD, n (%) | 45 (45.9) | 57 (46.7) | 0.90 |
| CVD, n (%) | 21 (21.4) | 24 (19.7) | 0.75 |
| ABI, ± SD | 0.40 ± 0.1 | 0.39 ± 0.1 | 0.20 |
| Rutherford II-4, n (%) | 50 (51.0) | 71 (58.2) | 0.29 |
| Rutherford III-5, n (%) | 48 (49.0) | 51 (41.8) | 0.29 |
| WIfI010, n (%) | 14 (14.3) | 21 (17.2) | 0.55 |
| WIfI020, n (%) | 28 (28.6) | 34 (27.9) | 0.91 |
| WIfI110, n (%) | 47 (48.0) | 60 (49.2) | 0.86 |
| WIfI120, n (%) | 9 (9.2) | 7 (5.7) | 0.33 |
| HbA1c, % (mmol/mol) ± SD | 8.8 (73) ± 1.5 | 8.9 (74) ± 1.5 | 0.71 |
| FBG, mg/dL ± SD | 116.1 ± 19.9 | 112.5 ± 19.6 | 0.19 |
| Total cholesterol, mg/dL ± SD | 205.3 ± 30.7 | 203.2 ± 28.2 | 0.60 |
| LDL cholesterol, mg/dL ± SD | 100.1 ± 18.4 | 101.3 ± 20.2 | 0.66 |
| Triglycerides, mg/dL ± SD | 215.0 ± 37.0 | 210.0 ± 36.8 | 0.31 |
| Creatinine, mg/dL ± SD | 1.5 ± 0.4 | 1.4 ± 0.4 | 0.17 |
| eGFR, mL/min/1.73m^2^ ± SD | 64.4 ± 15.4 | 64.4 ± 14.0 | 0.98 |
| Ca, mg/dL ± SD | 9.4 ± 0.8 | 9.6 ± 0.7 | 0.03 |
| Ph, mg/dL ± SD | 3.7 ± 0.8 | 3.7 ± 0.8 | 0.56 |
| Vitamin D, ng/mL ± SD | 48.0 ± 16.0 | 49.0 ± 16.0 | 0.67 |
| Klotho, pg/mL ± SD | 495.4 ± 183.9 | 329.1 ± 136.8 | <0.01 |
| FGF23 pg/mL ± SD | 56.1 ± 17.3 | 62.2 ± 17.3 | <0.01 |

Data are reported as means (standard deviation) for continuous variables and numbers (percentages) for categorical variables. BMI, Body Mass Index; CAD, Coronary Artery Disease; CVD, Cerebrovascular Disease; ABI, Ankle Brachial Index; WIfI, Wound, Ischemia, foot Infection; FBG, Fasting Blood Glucose; eGFR, estimated Glomerular Filtration Rate; Ca, Calcium; Ph, Phosphorus; FGF23, Fibroblast Growth Factor 23.

**Supplemental Figures**

**Supplemental Figure 1**


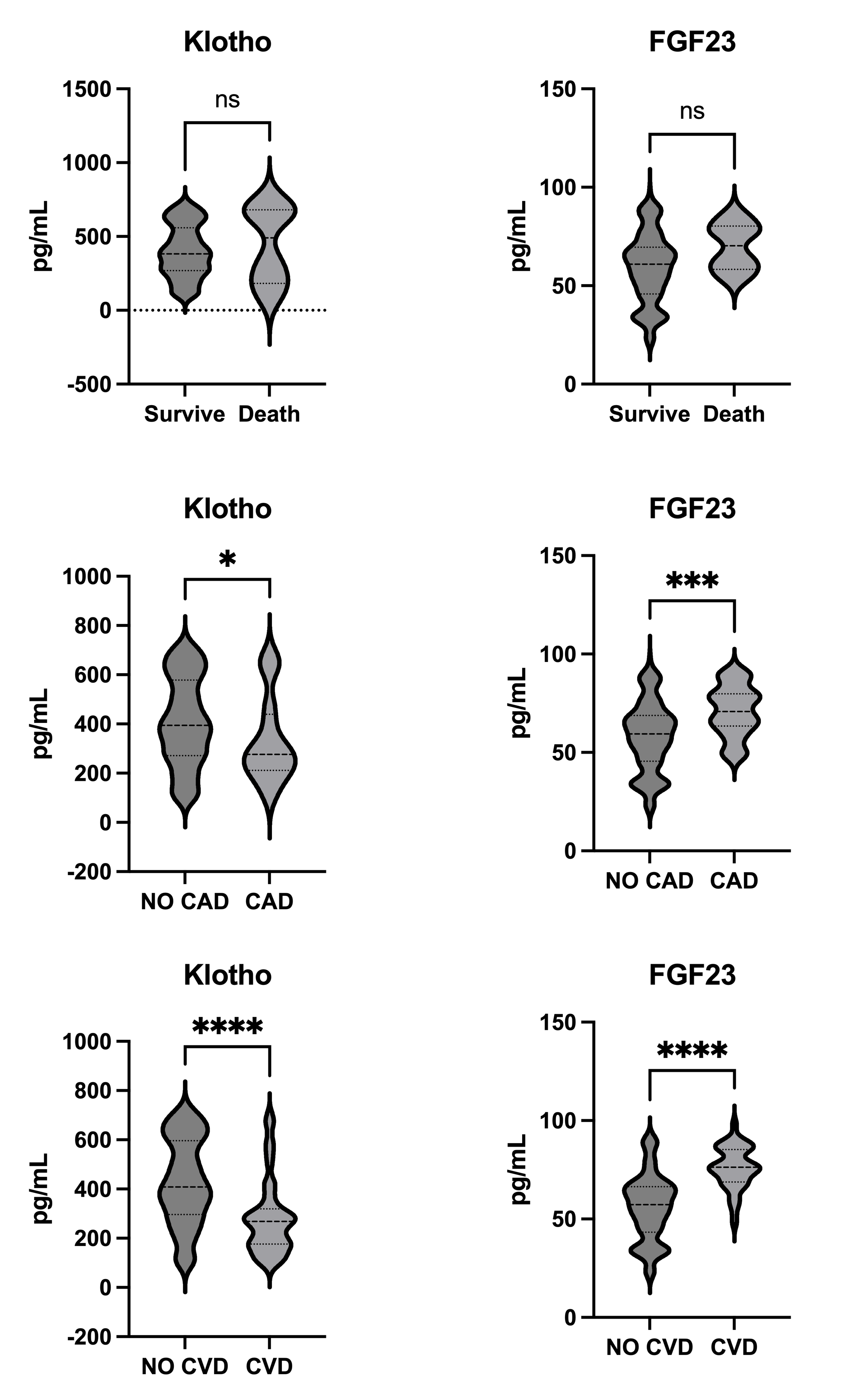


Klotho, and FGF23 levels according to cardiovascular death, coronary heart disease (CAD), and cerebrovascular disease (CVD) outcomes. On the violin plots, shape shows the distribution, central line represents the median, upper line represents the upper interquartile range (IQR) and the lower line represents the lower IQR. **** = p<0.0001, *** = p<0.001, * = p<0.05.

**Supplemental Figure 2**


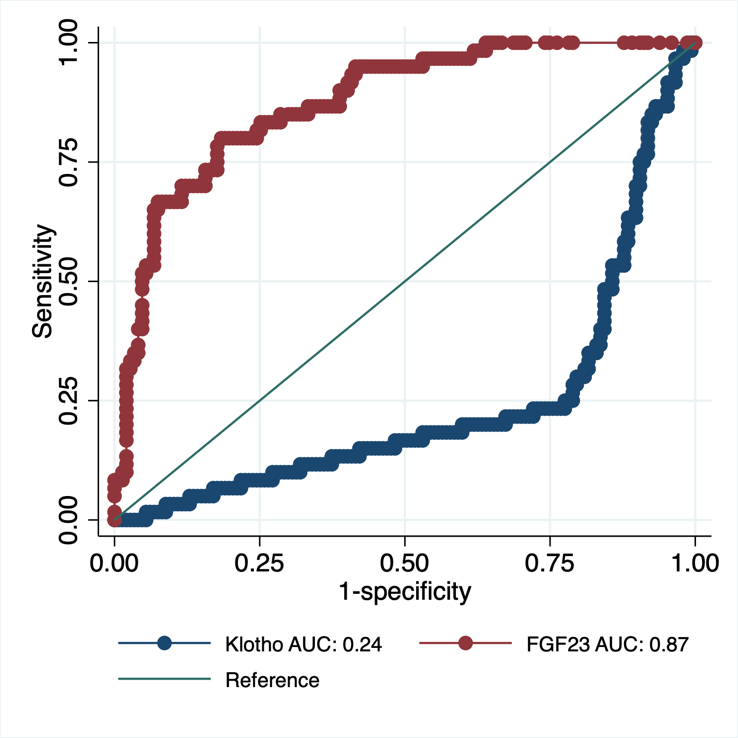

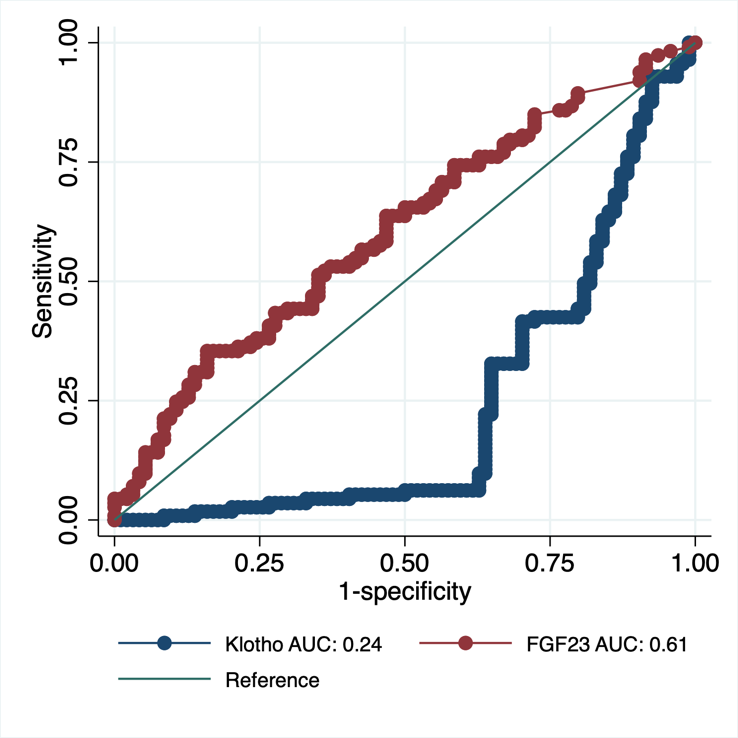


On the left, receiver operating characteristic (ROC) curve analysis to predict incidence of MACE related to Klotho, and FGF23 levels showing two areas under the curve (AUC). p<0.001.

On the right, Receiver operating characteristic (ROC) curve analysis to predict incidence of MALE related to Klotho, and FGF23 levels showing two areas under the curve (AUC). p<0.001.
